# Taking the leap toward human-specific nonanimal methodologies: The need for harmonizing global policies for microphysiological systems

**DOI:** 10.1016/j.stemcr.2023.11.008

**Published:** 2023-12-21

**Authors:** Surat Parvatam, David Pamies, Francesca Pistollato, Sonja Beken, Indumathi Mariappan, Adrian Roth, Monica Piergiovanni, Benoît G.C. Maisonneuve, Lorna Ewart, Abhijit Majumder, Prajakta Dandekar, Rahul Date, Kasturi Mahadik, Saravanan Thiyagarajan, Sandra Coecke

**Affiliations:** 1 Humane Society International, Hyderabad, India; 2 SCAHT – Swiss Centre for Applied Human Toxicology. University of Basel. Missionsstrasse 64, 4055 Basel, Switzerland; 3 Humane Society International, Rue Belliard 40, 1040 Bruxelles, Belgium; 4 Federal Agency for Medicines and Health Products, Brussels, Belgium; 5 Center for Ocular Regeneration, LV Prasad Eye Institute, Hyderabad, India; 6 F. Hoffmann-La Roche, Basel, Switzerland; 7 European Commission, Joint Research Centre (JRC), Ispra, Italy; 8 NETRI, Lyon, France; 9 Emulate Inc, 27 Drydock Avenue, Boston, MA, USA; 10 Indian Institute of Technology Bombay (IITB), Mumbai, India; 11 Institute of Chemical Technology, Mumbai, India; 12 Jai Research Foundation, Gujarat, India; 13 Centre for Predictive Human Model Systems, Atal Incubation Centre-Centre for Cellular and Molecular Biology (AIC-CCMB), Hyderabad, India; 14 Aurigene Oncology Limited, Bangalore, India

## Abstract

With a recent amendment, India joined other countries that have removed the legislative barrier toward the use of human-relevant methods in drug development. Here, global stakeholders weigh in on the urgent need to globally harmonize the guidelines toward the standardization of microphysiological systems. We discuss a possible framework for establishing scientific confidence and regulatory approval of these methods.

## Main text

### Introduction

In March 2023, the Indian government passed an amendment to the New Drugs and Clinical Trials (2023) whereby several human-specific nonanimal methodologies (NAMs), including cell-based assays, microphysiological systems (MPS), and computational modeling, are now included in the list of nonclinical testing methods, along with animal studies. MPS is an umbrella term that has been used for miniature human cell–based *in vitro* constructs, including, organoids, three-dimensional (3D) tissues, and organ-on-chip (OoC) models that aim to mimic human physiology. With this move, India joins several countries, including the United States, the European Union (EU), Canada, Brazil, South Korea, and Japan, that have made legislative strides toward NAMs. Removal of this legislative barrier provides incentives and confidence to the industry to accelerate the development and incorporation of these methods in their internal portfolio for decision making or through dynamic industry–academia partnerships.

An anonymous survey conducted by the International Consortium for Innovation and Quality in Pharmaceutical Development (also known as the IQ Consortium) among MPS suppliers and end users indicated the successful application of MPS models at various stages of drug development, including lead identification, preclinical safety, and drug efficacy (Beaurivage et al., 2019; Ewart et al., 2022; Foster et al., 2019; Marx, 2020).

MPS are also becoming an important instrument for informing the design of clinical trials. They can become a critical tool in cases such as rare or pediatric diseases, where it is often difficult to find adequate representation in clinical trials. Recently, a study investigated the cardiac liability associated with the polytherapy of repurposed drugs using cardiac MPS akin to a Phase I safety trial (Charrez et al., 2021).

Despite its promise and the increasing usage in academia and industry, the uptake of MPS globally has been slow for different reasons, including the lack of globally harmonized standards and performance-related issues (Ewart and Roth, 2021).

In a recent multistakeholder roundtable discussion including policymakers, regulatory bodies, researchers and industry stakeholders organized by the Centre for Predictive Human Model Systems, Atal Incubation Centre-Centre for Cellular and Molecular Biology (India), and Humane Society International/India, many of these impediments were raised and discussed. Many of these concerns are global, and stakeholders across the world need to work together to address them.

### The global standards for OoC and MPS devices

Standardization of performance metrics and the implementation of standard operating procedures are crucial factors in driving innovation and facilitating the widespread adoption of scientific methods. These standardization efforts recognize that the field will benefit from a diversity of human specific NAMs, which use different form factors and endpoints, but that drug developers and regulators need the tools to assess the performance and predictive validity of these models; standardization efforts therefore focus on the evaluation of mode quality and ensure that their use is consistent rather than focused on constraining model design. Several recent documents serve as valuable resources in this pursuit of promoting responsible reporting practices and refining experimental protocols (Hartung et al., 2019).

The Good Cell Culture Practice task force was established in 1989, and this subsequently led to the formulation of an initial guideline that has now been updated to incorporate new technologies, such as induced pluripotent stem cells and microphysiological systems including OoC technologies (Pamies et al., 2022). This effort has been aligned with the internationally recognized Economic Cooperation and Development (OECD) guidance document on Good In Vitro Method Practice.

The European Commission Joint Research Centre and the European standardization organizations CEN (European Committee for Standardization) and CENELEC (European Committee for Electrotechnical Standardization) organized the workshop “Putting Science into Standards” (Piergiovanni et al., 2021) to identify the needs of and priorities for the development of standards. To facilitate a complete characterization of OoC devices, a general analysis of the required technological/engineering characteristics and biological components was proposed. Standardization needs for biosciences include the characterization of cells and tissues, biomaterials, and extracellular matrix properties, as well as endpoints and reference compounds. The engineering aspects to be standardized were divided into sensing and integration, interoperability, and microfluidics (Piergiovanni et al., 2021). Such checklists can assist regulators in better determining the relevance and reliability of MPS devices.

In 2012, the National Center for Advancing Translational Sciences (NCATS) launched the Tissue Chip for Drug Screening program in collaboration with the NIH and the US Food and Drug Administration (FDA). This program has been instrumental in developing various high-throughput assays and 3D cellular microsystems that represent several organ systems, such as kidney and heart (MacQueen et al., 2018). This significant effort based on *in vitro* methods has demonstrated the vast potential of these methods within the regulatory framework. In 2016, NCATS partnered with the International Space Station National Laboratory to announce the Tissue Chips in Space project, in which tissue chips are being used to study the effect of microgravity on the human body. The project also announced awards for setting up Tissue Chip Testing Centers for independent testing of tissue chips to determine their functionality, reproducibility, robustness, and reliability.

### Framework for establishing scientific confidence and promote regulatory acceptance of MPS

One of the most critical aspects to be addressed to enhance regulatory acceptance of MPS devices is defining their Context of Use (CoU) and ensuring that they are fit for the purpose for which they are proposed (Box 1). MPS devices are usually designed starting from biomedical research questions; thus, it is crucial to define a CoU that fits in the regulatory decision making. In an OoC Organ-on-Chip In Development Strategy workshop conducted in 2019, four main CoUs were identified, including improving understanding of human disease mechanisms and etiology, predicting drug efficacy in humans, predicting drug toxicity in humans, paving the way to personalized (or precision) medicine (Mastrangeli et al., 2019).

Box 1 **Definitions of CoU and fit for purpose**

**Table:** 

Fit for purpose	An assessment of whether the method and the endpoints are suitable and adequate for the purpose for which the test was designed.
CoU	A description of the circumstances under which a 3Rs testing approach (replacement, reduction, and refinement of animals used in research, teaching, testing, and exhibition) is applied in the assessment of human or veterinary medicinal products and the limitations within which the available data adequately support the use of the 3Rs testing approach.

A general annotated toxicity test method template (ToxTemp) has been developed to fulfill the requirements listed in the OECD Guidance Document 211 (GD211), which describes method documentation for the purpose of safety assessment, as well as to guide end users on the types of answers and information required, the definition of acceptance criteria, and the level of cell model characterization. Such templates can assist both method developers to keep note of the required deliverables while designing the system and regulators in terms of what information would be required to make a regulatory decision (Krebs et al., 2019).

To test the predictive value of these test human-based systems, the ultimate reference data or the gold standard of the endpoints being assessed should be human data. These data could include human clinical, epidemiological, imaging, genomic, proteomic, or gene expression analysis data. However, in cases in which high-quality human data are not available, the comparison to animal data could be based on certain parameters (van der Zalm et al., 2022). The reproducibility of animal tests and its variance is the current baseline that regulators accept. This could be used to determine confidence intervals and performance benchmarks of data generated using NAMs. Recently, initial recommendations on how to evaluate data generated using a battery of *in vitro* assays suitable for developmental neurotoxicity have been provided. Performance benchmarks and a flexible framework could be used to assess the predictive capacity of NAMs (van der Zalm et al., 2022).

Data generated using MPS could also be compared to historical data generated using more simplistic *in vitro* models or animal test results (Ingber, 2022)—for example, by testing drugs that have been shown to be safe and effective in preclinical animal testing, but failed in clinical trials due to toxicity or lack of efficacy.

Because NAMs provide mechanistic insights on the molecular, cellular, and tissue mechanisms underlying drug or chemical effects, their human biological relevance can also be shown by the recapitulation of specific key events from an Adverse Outcome Pathway (AOP). AOPs are structured and mechanistic representations of how molecular, cellular, and organ-level key events lead to an adverse event upon exposure to a toxin/substance. An MPS does not need to cover all of the key events within an AOP to be useful. Data obtained through MPS could also be integrated with data provided by, for example, *in silico* tools, simple *in vitro* test systems, and read across, and contribute to the weight of evidence for safety and/or efficacy of medicines.

Accuracy can also be evaluated by testing NAMs against positive and negative reference compounds for which sufficient data are available. The IQ Consortium also frequently releases publication series that analyze reference compounds to assess specific MPS models. The EU Reference Laboratory for alternatives to animal testing library of reference chemicals is also a catalog of chemical lists that can be used to standardize, qualify, characterize, or compare *in vitro*, *in chemico*, and *in silico* methods and models.

There is a need for global guidelines that clearly address the requirements for the acceptance of results that are generated with a human-specific NAM for a particular CoU, including the description of the CoU and the qualification criteria, with a detailed list of references. One such example is the guideline on reproductive toxicology from the International Council for Harmonisation of Technical Requirements for Pharmaceuticals for Human Use (ICH S5(R3)). Acceptance of such frameworks across different regulatory bodies could help harmonize data standards and acceptance criteria for MPS. In addition, creating globally agreed-upon lists of data-rich reference compounds that can be used to support the qualification of an assay or a battery of assays within a particular CoU within drug development would be instrumental in harmonizing the qualification of MPS. In 2018, ICH brought together international regulatory agencies (e.g., US FDA, European Medicines Agency [EMA], Japan Pharmaceutical Manufacturers Association, Health Canada, Swissmedic) to develop Q&As (questions and answers) for the ICH E14 and ICH S7B guidelines that describe nonclinical and clinical risk assessment strategies to determine whether a new drug was associated with a risk of cardiac arrhythmia. This work was informed by the outcomes of the Comprehensive *in vitro* Proarrhythmia Assay, which had as its objective the improvement of the assessment of the proarrhythmic potential of new drugs by integrating three main nonclinical measurements: *in vitro* assays to assess the effect of drugs on human ventricular ion channels, *in silico* measurements to determine the net effect on cardiac action potential, and the use of integrated biological systems, such as stem cell–derived cardiac myocytes. This initiative has helped to develop best practices for the design, conduct, analysis, interpretation, and reporting of *in vitro*, *in silico*, and *in vivo* nonclinical assays for predicting possible arrhythmias by new molecules.

### A flexible regulatory pipeline

Lack of a clear pipeline for the data generated using human-specific NAMs can also deter the end users. Many countries, including the United States and the EU have designed specific routes for submission of these types of data. For example, the US FDA allows data submission through the Innovative Science and Technology Approaches for New Drugs Pilot Program from the Center for Drug Evaluation and Research.

Moreover, the EMA Innovation Task Force, a multidisciplinary group that includes scientific, regulatory, and legal competences that ensures coordination across the EMA and provides a forum for early dialog on innovative aspects and tools in medicines development, now specifically covers the regulatory acceptance of NAMs, in close collaboration with the EMA’s 3Rs ((replacement, reduction, refinement) Working Party, to foster their integration in the development and evaluation of medicines.

The EMA, in its guideline on the principles of regulatory acceptance of 3Rs testing, allows for the voluntary submission of data generated through NAMs, such as MPS, along with data generated through existing methods. These data are not used to make product-based regulatory decisions during this period; however, this approach allows gathering sufficient information on an NAM before considering its possible future regulatory acceptance. Such an approach could be used globally at the early stages of NAM development, to build confidence in these methods.

### Conclusions

The success and effectiveness of MPS will be determined in part by their ease of use, the availability of off-the-shelf solutions, their functionality over time, understanding to what extent they are fit for purpose within a particular CoU, how reliable they are in accurately recapitulating human and animal systems biology, and whether their use can be standardized for decision making and implementing advanced global policies.

Funding retrospective studies aimed at comparing tools to determine why some models are more successful than others and establishing a system for institutional learning are considered reasonable proposals to improve model validity and trust (Scannell et al., 2022). True qualification studies are lengthy and expensive, and the funding of prospective studies to assist in the generation of the needed trust-building data, as required by regulatory authorities, is also crucial.

Ultimately, the implementation of human-specific NAMs for regulatory purposes should preferably be harmonized at a global level. This could be achieved by international organizations, such as the ICH or the OECD, which bring together regulatory authorities and industry representatives to develop specific guidelines in various sectors, such as pharmaceuticals, industrial chemicals, pesticides, and personal care products. Such international and multistakeholder conversations would be critical and assist in prioritizing CoUs and harmonize views on qualification requirements. Iteratively building upon successful case studies would ultimately lead to wider adoption and implementation.
